# Quantitative susceptibility mapping using multi-channel convolutional neural networks with dipole-adaptive multi-frequency inputs

**DOI:** 10.3389/fnins.2023.1165446

**Published:** 2023-06-13

**Authors:** Wenbin Si, Yihao Guo, Qianqian Zhang, Jinwei Zhang, Yi Wang, Yanqiu Feng

**Affiliations:** ^1^School of Biomedical Engineering, Southern Medical University, Guangzhou, China; ^2^Guangdong Provincial Key Laboratory of Medical Image Processing and Guangdong Province Engineering Laboratory for Medical Imaging and Diagnostic Technology, Southern Medical University, Guangzhou, China; ^3^Department of Radiology, Hainan General Hospital (Hainan Affiliated Hospital of Hainan Medical University), Haikou, Hainan, China; ^4^Department of Biomedical Engineering, College of Engineering, Cornell University, Ithaca, NY, United States; ^5^Department of Radiology, Weill Cornell Medicine, Cornell University, New York, NY, United States; ^6^Guangdong-Hong Kong-Macao Greater Bay Area Center for Brain Science and Brain-Inspired Intelligence and Key Laboratory of Mental Health of the Ministry of Education, Southern Medical University, Guangzhou, China

**Keywords:** quantitative susceptibility mapping, magnetic resonance imaging, deep learning, convolutional neural networks, image processing

## Abstract

Quantitative susceptibility mapping (QSM) quantifies the distribution of magnetic susceptibility and shows great potential in assessing tissue contents such as iron, myelin, and calcium in numerous brain diseases. The accuracy of QSM reconstruction was challenged by an ill-posed field-to-susceptibility inversion problem, which is related to the impaired information near the zero-frequency response of the dipole kernel. Recently, deep learning methods demonstrated great capability in improving the accuracy and efficiency of QSM reconstruction. However, the construction of neural networks in most deep learning-based QSM methods did not take the intrinsic nature of the dipole kernel into account. In this study, we propose a dipole kernel-adaptive multi-channel convolutional neural network (DIAM-CNN) method for the dipole inversion problem in QSM. DIAM-CNN first divided the original tissue field into high-fidelity and low-fidelity components by thresholding the dipole kernel in the frequency domain, and it then inputs the two components as additional channels into a multichannel 3D Unet. QSM maps from the calculation of susceptibility through multiple orientation sampling (COSMOS) were used as training labels and evaluation reference. DIAM-CNN was compared with two conventional model-based methods [morphology enabled dipole inversion (MEDI) and improved sparse linear equation and least squares (iLSQR) and one deep learning method (QSMnet)]. High-frequency error norm (HFEN), peak signal-to-noise-ratio (PSNR), normalized root mean squared error (NRMSE), and the structural similarity index (SSIM) were reported for quantitative comparisons. Experiments on healthy volunteers demonstrated that the DIAM-CNN results had superior image quality to those of the MEDI, iLSQR, or QSMnet results. Experiments on data with simulated hemorrhagic lesions demonstrated that DIAM-CNN produced fewer shadow artifacts around the bleeding lesion than the compared methods. This study demonstrates that the incorporation of dipole-related knowledge into the network construction has a potential to improve deep learning-based QSM reconstruction.

## 1. Introduction

Magnetic susceptibility is an intrinsic local tissue property that describes the degree of being magnetized in an external magnetic field B0. Quantitative susceptibility mapping (QSM) (de Rochefort et al., 2010) generates the spatial distribution of magnetic susceptibility from the ΔB0 field created by tissue magnetization and is measured using magnetic resonance imaging (MRI) (Schweser et al., 2013; Wang and Liu, 2015). The ΔB0 field is generated by a magnetic dipole moment at a source location according to Maxwell's equations in vacuum. The contributors to tissue magnetic susceptibility include biometals and molecules, e.g., iron (Liu et al., 2010; Schweser et al., 2011; Langkammer et al., 2012; Ghassaban et al., 2019), calcium (Chen et al., 2018; Guo et al., 2019a,b), lipids (Dibb et al., 2017), and myelin (Lee et al., 2021). QSM has been widely applied for the diagnosis and evaluation of intracranial hemorrhage (Chen et al., 2014b; Sun et al., 2018), brain aging (Bilgic et al., 2012; Zhang et al., 2018; Vinayagamani et al., 2021), Alzheimer's disease (Kim et al., 2017; O'Callaghan et al., 2017; Gong et al., 2019; Tiepolt et al., 2020; Cogswell et al., 2021), Parkinson's disease (Acosta-Cabronero et al., 2017; He et al., 2021), Huntington's disease (Chen L. et al., 2019; Pagnozzi et al., 2019; Ravanfar et al., 2021), and multiple sclerosis (Chen et al., 2014a; Barkhof and Thomas, 2018). Moreover, a QSM image can clearly show the anatomical structure of deep brain nuclei, and thus, it has been used for accurate target localization in deep brain stimulation (Wang and Liu, 2015).

Although QSM demonstrates great potential for clinical applications, accurate QSM reconstruction is still a challenging problem. The division by near-zero values in the k-space of the dipole kernel in the dipole inversion step results in an amplification of noise and artifacts (Wang et al., 2013; Haacke et al., 2015; Wang and Liu, 2015). Multiple orientation sampling with respect to the B0 field and reconstruction with the calculation of susceptibility through multiple orientation sampling (COSMOS) can obtain the accurate susceptibility map (Liu et al., 2009), serving as a gold standard for the single-orientation QSM method. However, multiple orientation scans increase the burden in head positioning and extremely prolong the acquisition time, and thus, it is impractical for clinical application (Wharton and Bowtell, 2010). A variety of numerical stabilization methods have been developed to regularize the susceptibility reconstruction from single-orientation QSM data. Truncated k-space deconvolution (TKD) (Wharton et al., 2010; Tang et al., 2013), improved sparse linear equation and least squares (iLSQR) (Li et al., 2015), and streaking artifact reduction for QSM (STAR-QSM) methods (Wei et al., 2015) were proposed for the suppression of streaking artifacts in QSM reconstruction. In addition, various regularization algorithms that enforce certain prior information have been developed to improve QSM reconstruction (Liu et al., 2011; Liu T. et al., 2012; Bao et al., 2016; Chatnuntawech et al., 2017; Wen et al., 2021). For example, the morphology-enabled dipole inversion (MEDI) constrains the reconstructed QSM map to have similar edges as in the magnitude image (Liu et al., 2011; Liu J. et al., 2012; Liu T. et al., 2012).

Recently, convolutional neural networks (CNN) were introduced to QSM reconstruction and demonstrated higher accuracy compared with the non-learning-based methods (Kyong Hwan et al., 2017; Yoon et al., 2018; Bollmann et al., 2019; Chen et al., 2020; Jung et al., 2020b; Zhang et al., 2020; Feng et al., 2021; Gao et al., 2021). DeepQSM (Bollmann et al., 2019), QSMnet (Yoon et al., 2018), and QSMnet+ (Jung et al., 2020b) adopted the popular Unet (Ronneberger et al., 2015) to approximate the dipole inversion from the tissue field to the susceptibility map. However, the network construction in these methods did not consider the intrinsic nature of dipole kernel (Jung et al., 2020a,b). Recently, the model-driven deep learning methods, where the network was constructed by unrolling the conventional iterative model-based algorithms, were incorporated to solve the dipole inversion problem in QSM. VaNDI trained a variational network to optimize the parameters in an unrolled gradient descent algorithm for dipole inversion (Polak et al., 2020), and MoDL-QSM combined the physical model of susceptibility tensor imaging with CNN (Feng et al., 2021). Physics modeling can be used to reduce generalization errors or improve fidelity of deep learning (Zhang et al., 2020).

Considering that streaking artifacts are directly related to small values in the frequency response of the dipole kernel, we proposed a novel dipole kernel-adaptive multi-channel CNN (DIAM-CNN) for the dipolar inversion from single-orientation QSM data. DIAM-CNN divided the original tissue field into high-fidelity and low-fidelity components based on the frequency response of dipole kernel and inputs the two components together with the original tissue field as three individual channels to a multi-channel Unet. The proposed network was trained and validated on *in vivo* patient data using COSMOS results as a reference.

## 2. Materials and methods

### 2.1. Datasets

#### 2.1.1. Data of healthy volunteers

The dataset of 12 healthy volunteer scans from QSMnet (Yoon et al., 2018) was enrolled in this study. The volunteers were scanned with a 3D single-echo gradient-recalled sequence (at 3T; TIM Trio MRI was used in nine datasets, and MAGNETOM Skyra was used in three datasets. TE = 25 ms, TR = 33 ms, 1 mm isotropic resolution, flip angle = 15^o^, bandwidth = 100 Hz/pixel, acceleration factor = 2 × 2, the total acquisition time = 5 min 46 s). Each subject was scanned with five head orientations, and the multiple orientation data were reconstructed using COSMOS to obtain a reference susceptibility map.

#### 2.1.2. Data of intracerebral hemorrhage

A patient with intracerebral hemorrhage (ICH) was enrolled. The hemorrhage patient data were acquired using a 3T system (HDx, GE Healthcare, Waukesha, WI, USA), and scan parameters included flip angle = 15^o^, first TE = 5 ms, ΔTE = 4.6 ms, number of echoes = 6, acquisition matrix = 184 × 210 × 144, and 1 mm isotropic resolution. iLSQR was performed to reconstruct a susceptibility map (Li et al., 2015). IRB-approved written consent was signed by each participant.

##### 2.1.2.1. Simulation of QSM data with high susceptibility region

The hemorrhage lesion was manually delineated from the susceptibility map reconstructed by iLSQR. After that, this lesion region with high susceptibility was overlapped with one of the reference susceptibility maps from the multiple orientation data. Finally, the susceptibility map was convolved with the dipole kernel and then added with Gaussian noise to generate the corresponding tissue field. All subjects signed IRB-approved written consent forms.

### 2.2. Architecture of the DIAM-CNN network

**Figure 1:**
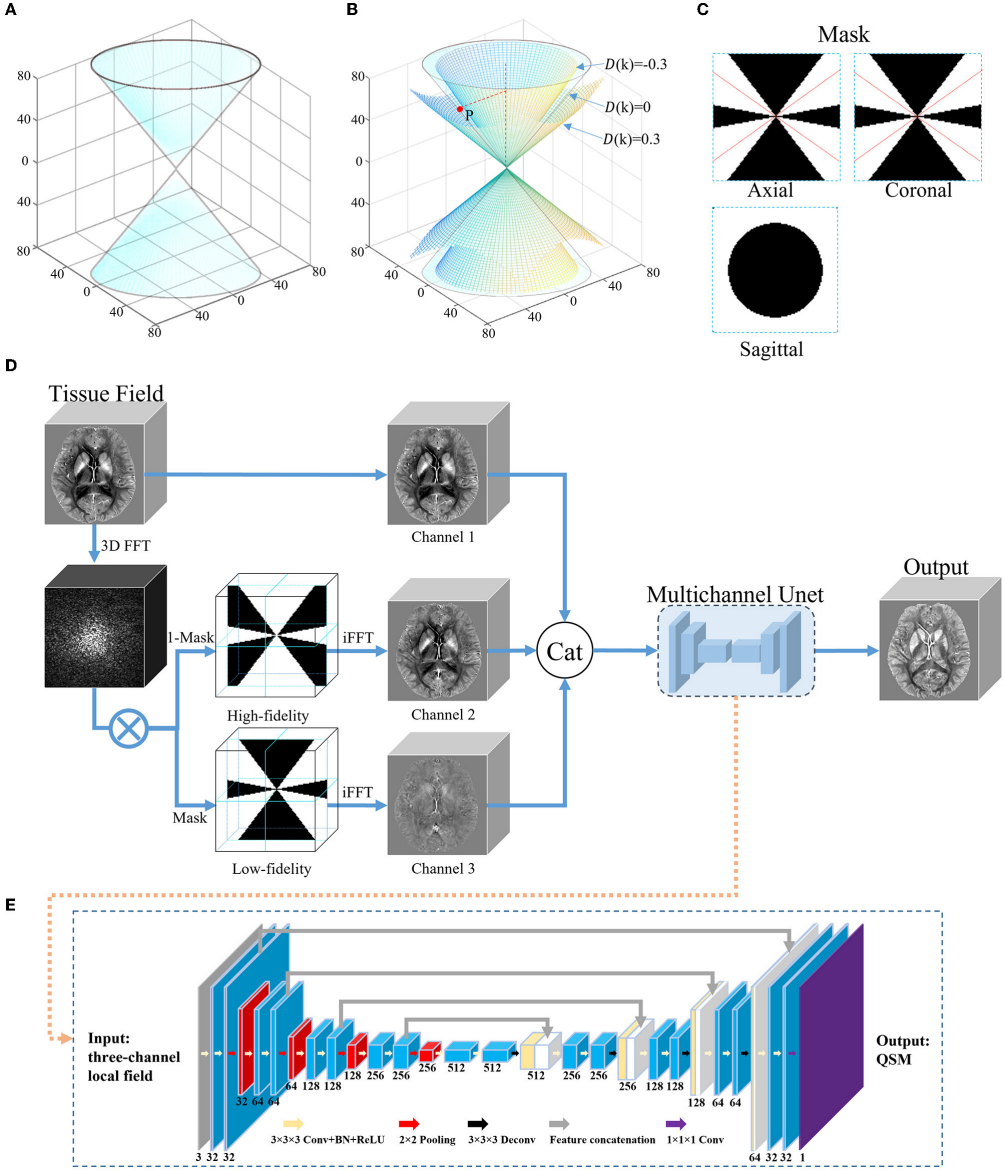
An overview of the proposed DIAM-CNN method. **(A)** Conical surface of *D*(*k*) = 0 in the frequency domain of the dipole kernel. **(B)** The conical surfaces of *D*(*k*) = 0.3, −0.3, and 0, and a representative point *P* on the surface of *D*(*k*) = 0. **(C)** Three orthogonal planes of the mask for the low-fidelity component generated with a representative threshold of 0.3 (|*D*(*k*)| ≤ 0.3). The red lines correspond to the conical surface of *D*(*k*) = 0. **(D)** Network architecture of DIAM-CNN. DIAM-CNN divided the tissue field into low-fidelity and high-fidelity components using the mask in **(C)**, and then, it inputs the two components as additional channels into a multichannel Unet. **(E)** Schematic diagram of the multichannel Unet. The network was designed with 18 convolutional layers (kernel size = 3 × 3 × 3), one convolutional layer (kernel size = 1 × 1 × 1), four max pooling layer strides (kernel size = 2 × 2 × 2), four transposed convolution layer strides (kernel size = 3 × 3 × 3), and four feature concatenations.

In theory, the tissue field can be expressed as a convolution of the susceptibility distribution with a dipole kernel in spatial domain (Wang and Liu, 2015). This convolution corresponds to multiplication in the frequency domain as follows:


(1) $$\begin{array}{l} B\left(k\right)\;=\;D\left(k\right)X\left(k\right)\;+\;N\left(k\right), \end{array}$$

where *B*(*k*), *D*(*k*), *X*(*k*), and *N*(*k*) are tissue field, dipole kernel, magnetic susceptibility, and noise in the frequency domain, respectively. According to Eq. (1), the tissue field can be considered as filtering the susceptibility map by the dipole with a frequency response of *D*(*k*). In the frequency domain, the susceptibility multiplied by the large *D*(*k*) magnitude values is preserved with a high fidelity, and the susceptibility multiplied by the small *D*(k) magnitude values is preserved with a low fidelity. In an extreme case, the information of *X*(*k*) was lost on positions where *D*(*k*) = 0. The dipole kernel in the frequency domain can be expressed by


(2) $$\begin{array}{l} D\left(k\right)\;=\;1/3-\frac{{k_{z}}^{2}}{{k_{x}}^{2}+{k_{y}}^{2}+{k_{z}}^{2}}, \end{array}$$

where *k*_*x*_, *k*_*y*_, and *k*_*z*_ are k-space vectors in three orthogonal directions. *D*(*k*) ranges from −2/3 to 1/3. Figure 1A shows the conical surface of *D*(*k*) = 0 in the frequency domain of the dipole kernel. The reconstruction of QSM requires the following operation:


(3) $$\begin{array}{l} \hat{X\left(k\right)}\;=\;B\left(k\right)/D\left(k\right)\;=\;X\left(k\right)\;+\;N\left(k\right)/D\left(k\right). \end{array}$$

The error in the above inversion is mainly from the noise amplification due to the division by small *D*(*k*) values. Thresholding the dipole in the frequency domain can be used to divide the tissue field into low-fidelity and high-fidelity components. Figure 1B shows the conical surfaces of *D*(*k*) at a representative threshold of 0.3 and a representative point *P* on the surface where *D*(*k*) = 0. The magnitude of *D*(*k*) is equal to or less than 0.3 in the region between the two conical surfaces and >0.3 in the residual regions. Figure 1C shows three orthogonal planes of the mask for the low-fidelity component (|*D*(*k*)| ≤ 0.3). The red lines correspond to the conical surface of *D*(*k*) = 0.

The network architecture of the DIAM-CNN is shown in Figures 1D, E. First, three-dimensional Fourier transform was performed to transform the tissue field to the frequency domain. Second, the tissue field was divided into high-fidelity and low-fidelity components using the mask shown in Figure 1C. Third, the tissue field was converted to the spatial domain by using inverse Fourier transform. Finally, the original tissue field, high-fidelity component, and low-fidelity component were concatenated and input to a multi-channel Unet network as three individual channels.

DIAM-CNN adopted a multichannel 3D Unet architecture (Chen Y.-C. et al., 2019; Souza et al., 2020). First, there were five blocks in the encoder component of the network. Each encoder block involved two 3 × 3 × 3 convolutional layers. The first layer had 32 channels, and the channel number was doubled in each layer compared to the previous layer. Batch normalization (Ioffe and Szegedy, 2015) and rectified linear unit (Glorot et al., 2011) were applied after each convolutional layer. Then, a 2 × 2 × 2 max pooling layer was performed after each block. Second, there were four blocks in the decoder component. Each decoder block consisted of 3 × 3 × 3 transposed convolutional layers and the corresponding feature concatenation. Each concatenation was followed by two convolutional layers. Finally, a 1 × 1 × 1 convolutional layer was used in the last layer.

The physical model loss (model loss), voxel-based difference (L1 loss), and edge-based difference (gradient loss) in QSMnet were used (Yoon et al., 2018). The total loss was defined as a weighted sum of the model, L1, and gradient losses with experientially determined weightings of 0.5, 1, and 0.1, respectively.

The “ADAM” optimizer (Kingma and Ba, 2014) was used to optimize the weights of the network during training. The initial learning rate was set at 0.001 and then decayed by 0.1 time when the improvement was not obvious. The batch size was set 8, and the training process was stopped at 160 epochs.

### 2.3. Network training and testing

The DIAM-CNN network was implemented using Python 3.6 and trained using one NVIDIA TITAN X (Pascal) GPU card. To fit into GPU memory, the patch size for the DIAM-CNN training was cropped to 64 × 64 × 64. The patch was generated with an overlapping scheme of 66% overlap. To improve training efficiency, patches with less than 10% tissue region were discarded.

The 12 healthy subjects with five orientations were divided into five for training, two for validation, and five for testing. In addition, rotation was applied to the COSMOS susceptibility map and the tissue field map to increase the diversity of orientation in the training dataset. The rotation angle with −15^o^ and 15^o^ relative to B0 was chosen. The trained DIAM-CNN network is available at https://github.com/SMU-BME-MRI-LIST/DIAM-CNN.

### 2.4. Performance evaluation

To demonstrate that DIAM-CNN enhanced the quality of QSM maps, the five healthy volunteer test sets were processed using QSMnet and DIAM-CNN. In addition, MEDI and iLSQR QSM maps were also reconstructed for comparison. In MEDI reconstruction, lambda was set to 3,000 (Yoon et al., 2018). With the COSMOS reconstruction as a reference, the following metrics were calculated for quantitative evaluation of QSM algorithms: high-frequency error norm (HFEN), peak signal-to-noise-ratio (PSNR), normalized root mean squared error (NRMSE), and the structure similarity index measure (SSIM) (Langkammer et al., 2018). Overall error, high-frequency deviation, noise, and “visual” fidelity were the objectives of RMSE, HFEN, PSNR, and SSIM, respectively.

To further demonstrate the quantitative accuracy of the DIAM-CNN in deep gray matters, we performed a linear regression analysis of the susceptibility values between COSMOS and deep learning-based methods in the putamen (PUT) and globus pallidus (GP). The linear regression line and R^2^ value were calculated between the susceptibility values of COSMOS and those of QSMnet and DIAM-CNN. In addition, for the QSM of the brain with a simulated high-susceptibility hemorrhage region, the susceptibility map reconstructed by iLSQR, QSMnet, and DIAM-CNN was compared by visual inspection. For the purpose of locating and assessing the variance between method-specific QSM results and the reference, visual inspections were carried out. The visual inspections and anatomical ROI evaluation in this study were supplied by two radiologists with extensive expertise.

## 3. Results

We evaluated the performance of DIAM-CNN with varying dipole thresholds. As shown in Table 1, the highest PSNR (PSNR = 43.15 ± 1.19), the smallest NRMSE (NRMSE = 51.79 ± 3.74), and the highest SSIM (SSIM = 0.909 ± 0.011) were approximately achieved at a threshold of 0.3. HFEN approximately reached the minimum at a threshold of 0.4 (HFEN = 62.13 ± 8.93). Thus, the threshold of 0.3 was used in all the following implementations of DIAM-CNN. We also evaluated the performance of DIAM-CNN with two input channels by selecting any two out of the three channels. As shown in Table 2, the three-channel DIAM-CNN outperformed all the two-channel DIAM-CNN in terms of HFEN, NRMSE, PSNR, and SSIM.

**Table 1:** Mean and standard deviation of the quantitative performance metrics, namely, HFEN, PSNR, NRMSE, and SSIM for DIAM-CNN with different dipole thresholds.

**Dipole-threshold**	**HFEN (%)**	**NRMSE (%)**	**SSIM**	**PSNR**
±0.1	65.55 ± 9.66	53.29 ± 3.78	0.905 ± 0.012	42.90 ± 1.18
±0.2	62.35 ± 8.89	51.81 ± 3.60	0.909 ± 0.011	43.15 ± 1.20
±0.3	62.40 ± 8.90	**51.79** **±** **3.74**	**0.909** **±** **0.011**	**43.15** **±** **1.19**
±0.4	**62.18** **±** **9.13**	52.47 ± 3.87	0.908 ± 0.011	43.04 ± 1.18
±0.5	63.04 ± 9.23	52.65 ± 3.81	0.906 ± 0.011	43.00 ± 1.19

The bold part indicates the optimal value of each metric.

**Table 2:** Mean and standard deviation of the quantitative performance metrics, namely, HFEN, PSNR, NRMSE, and SSIM for DIAM-CNN with varying channels (1, original tissue field; 2, high-fidelity component; 3, low-fidelity component).

**Channels**	**HFEN (%)**	**NRMSE (%)**	**PSNR**	**SSIM**
1 and 2	64.90 ± 9.12	53.07 ± 3.72	42.95 ± 1.18	0.906 ± 0.011
1 and 3	65.68 ± 9.68	53.50 ± 3.80	42.87 ± 1.18	0.905 ± 0.011
2 and 3	65.59 ± 9.07	53.68 ± 3.39	42.84 ± 1.16	0.901 ± 0.011
1, 2 and 3	**62.40** **±** **8.90**	**51.79** **±** **3.74**	**43.15** **±** **1.19**	**0.909** **±** **0.011**

The bold part indicates the optimal value of each metric.

**Figure 2:**
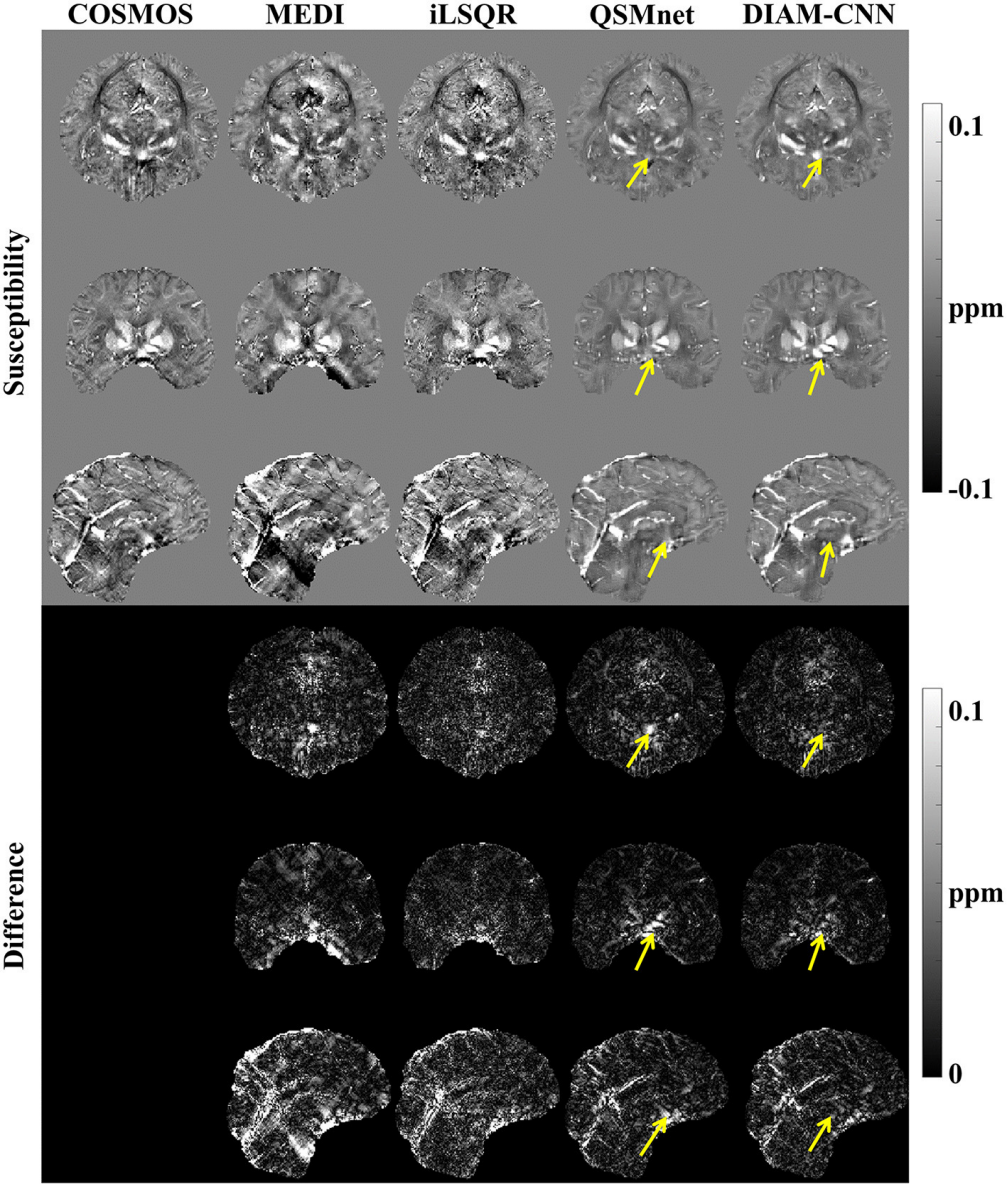
Comparison of QSM of one subject reconstructed using the non-learning-based dipole inversion algorithms (MEDI and iLSQR) and QSMnet and DIAM-CNN. Row 1, 2, and 3: axial view, coronal view, and sagittal view. Row 4, 5, and 6: the error map in each direction. Yellow arrows indicate regions with an obvious difference between QSMnet and DIAM-CNN.

**Figure 3:**
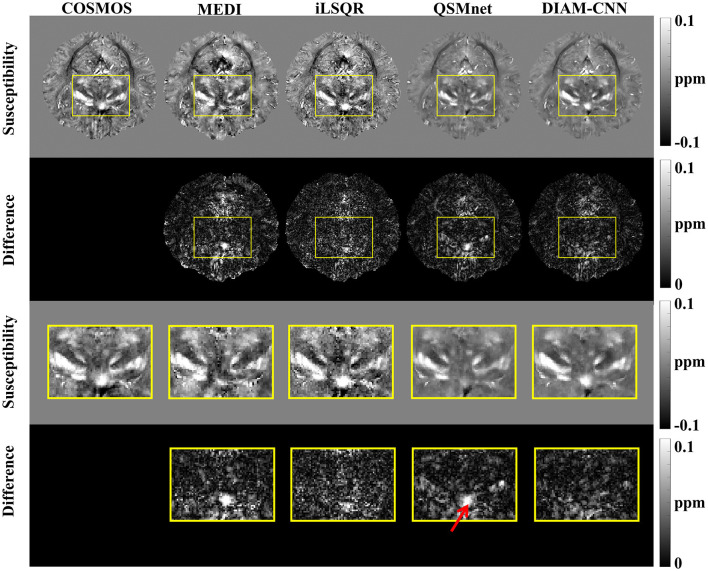
An axial view of the QSM map **(first row)** and the corresponding difference maps **(second row)** of the subject in Figure 2. Zoomed-in view of the map shown in the last two rows [**(third row)** and **(fourth row)**]. DIAM-CNN shows smaller errors relative to the COSMOS reference than the compared methods. The red arrow points at the subcallosal cingulate gyrus region where the QSMnet result has obvious underestimation, while the DIAM-CNN result is accurate.

The QSM maps and the corresponding error maps from a representative test dataset reconstructed by different methods are displayed in Figure 2. Starting from the first column, the method for QSM reconstruction is COSMOS. As against the non-learning-based QSM dipole inversion algorithms (MEDI and iLSQR) in the second and third columns, the visual comparison of the results of QSMnet and DIAM-CNN was shown in columns 4 and 5. DIAM-CNN yielded fewer streaking artifacts compared to the non-learning-based methods. As shown by the yellow arrows, DIAM-CNN produced a more accurate susceptibility map compared to the QSMnet baseline. Zoomed-in views of the axial plane of Figure 2 are displayed in Figure 3. The DIAM-CNN map was less noisy than those of MEDI and iLSQR. As the last row shows, in comparison to QSMnet, DIAM-CNN yielded lower residuals in certain brain regions, including the nucleus accumbens septi, gyrus rectus, caudate nucleus, putamen (PUT), and globus pallidus (GP). In the subcallosal cingulate gyrus region, as pointed out by the red arrow, QSMnet yielded obvious underestimation, while DIAM-CNN revealed almost identical results to that of COSMOS.

**Figure 4:**
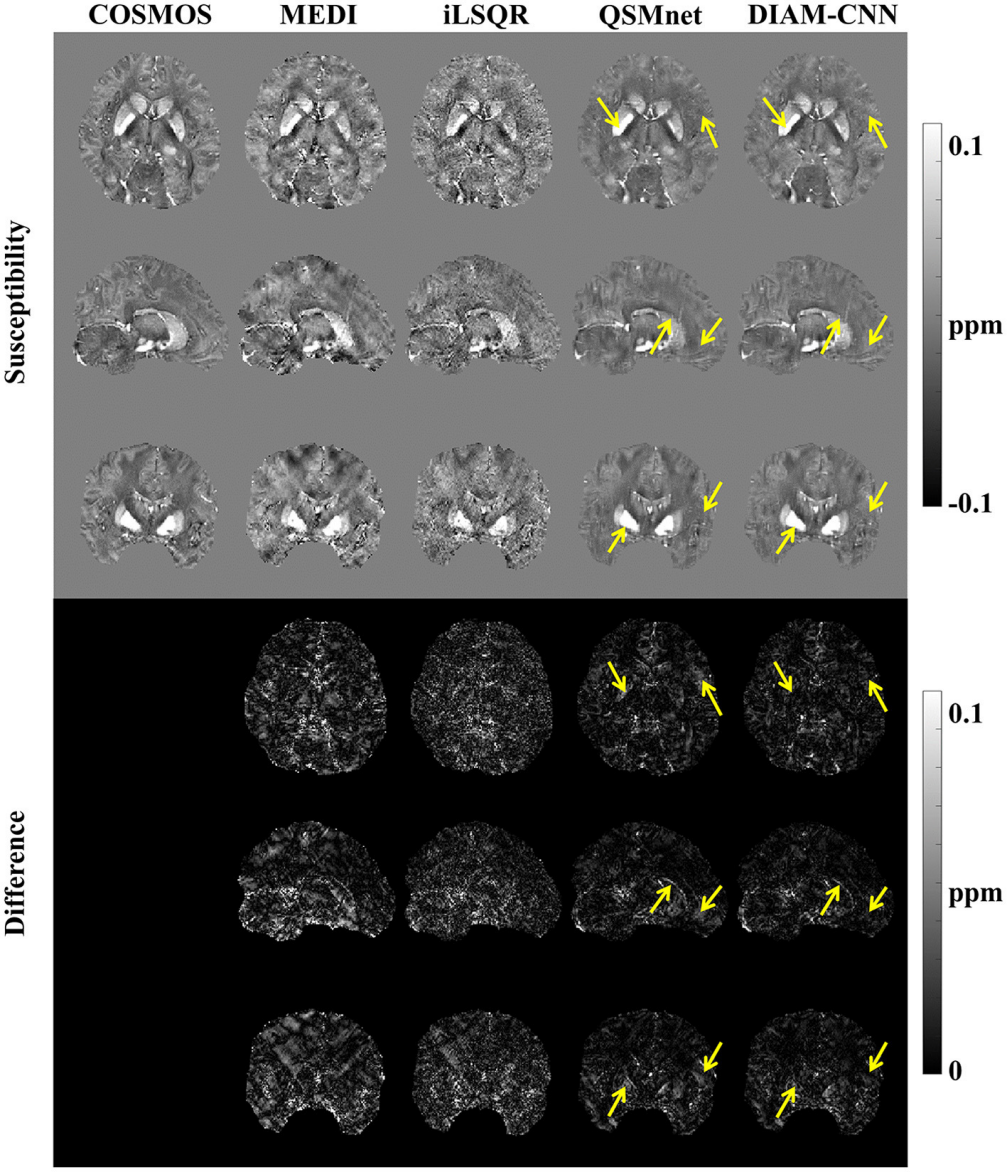
Comparison of QSM of another subject reconstructed using the non-learning-based dipole inversion algorithms (MEDI and iLSQR) and QSMnet and DIAM-CNN. Row 1, 2, and 3: axial view, coronal view, and sagittal view. Row 4, 5, and 6: the error map in each direction. Yellow arrows indicate regions with an obvious difference between QSMnet and DIAM-CNN.

**Figure 5:**
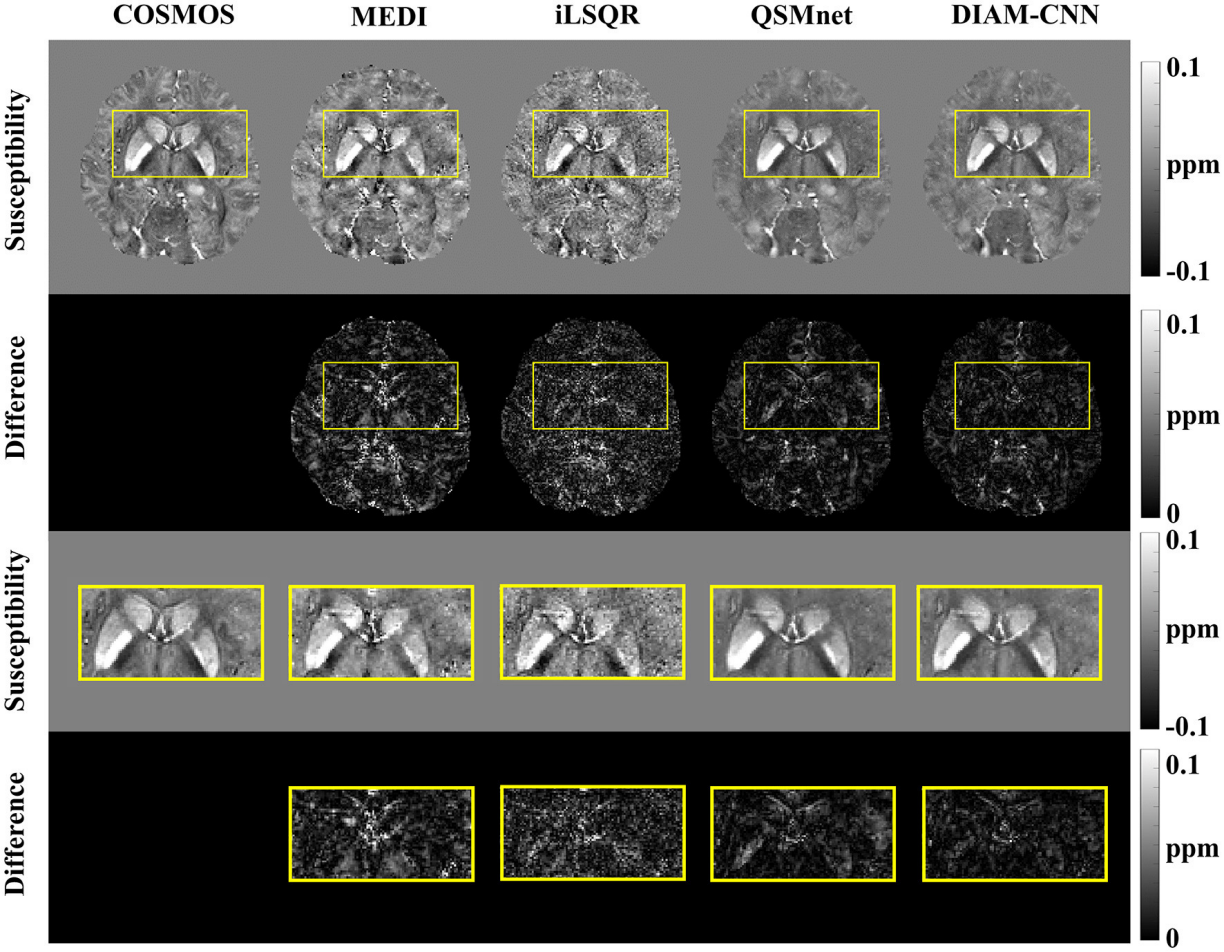
An axial view of the QSM map **(first row)** and the corresponding difference maps **(second row)** of the subject in Figure 4. Zoomed-in view of the map shown in the last two rows [**(third row)** and **(fourth row)**]. DIAM-CNN shows smaller errors relative to the COSMOS reference than the compared methods.

Figure 4 shows QSM slices from another test subject generated from our DIAM-CNN compared with MEDI, iLSQR, and QSMnet. Zoomed-in views of the axial plane of Figure 4 are displayed in Figure 5. Compared to QSMnet, DIAM-CNN produced fewer errors in regions including the caudate nucleus, the putamen, and the telencephalic white matter.

The quantitative HFEN, PSNR, NRMSE, and SSIM metrics calculated using the five test subjects was presented in Table 3. Compared to MEDI and iLSQR, learning-based dipole inversion algorithms including QSMnet and DIAM-CNN achieved higher PSNR (the higher the better) and lower NRMSE (the lower the better), while iLSQR obtained the best HFEN among these methods and MEDI achieved the best SSIM. DIAM-CNN outperformed QSMnet in all four criteria.

**Table 3:** Mean and standard deviation of the quantitative performance metrics, namely, HFEN, PSNR, NRMSE, and SSIM for the four reconstruction methods (MEDI, iLSQR, QSMnet, and DIAM-CNN).

**Methods**	**HFEN (%)**	**NRMSE (%)**	**PSNR**	**SSIM**
MEDI	68.68 ± 4.37	92.85 ± 7.70	38.10 ± 1.04	**0.956** **±** **0.017**
iLSQR	**61.31** **±** **5.71**	72.08 ± 6.17	40.29 ± 0.99	0.883 ± 0.033
QSMnet	63.73 ± 9.81	53.07 ± 3.92	42.94 ± 1.19	0.906 ± 0.012
DIAM-CNN	62.40 ± 8.90	**51.79** **±** **3.74**	**43.15** **±** **1.19**	0.909 ± 0.011

The bold part indicates the optimal value of each metric.

**Figure 6:**
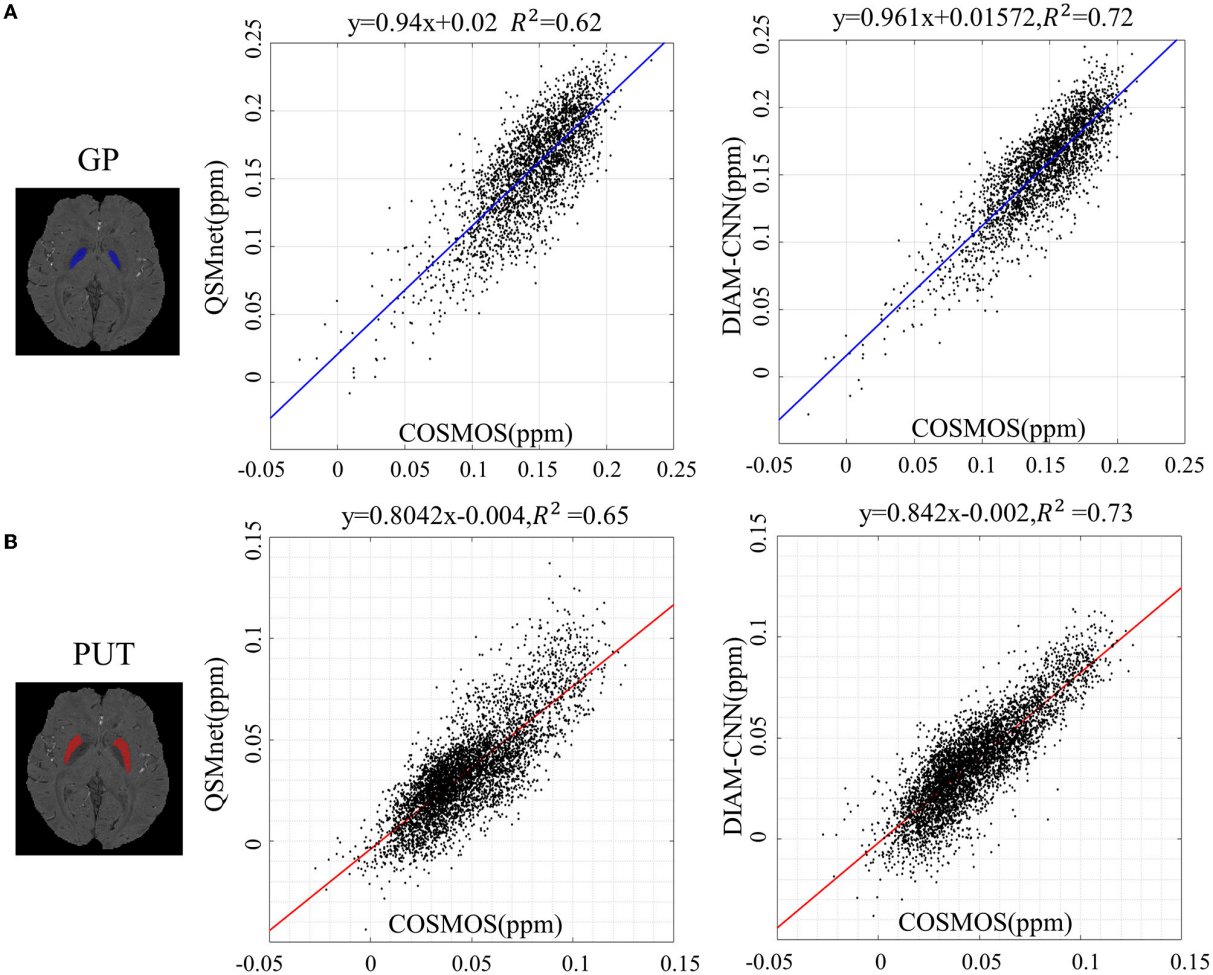
Linear regression of the susceptibility values in the globus pallidus **(A)** and the putamen **(B)** between COSMOS and deep learning-based methods (QSMnet and DIAM-CNN).

Linear correlation diagrams with the COSMOS result as the transverse axis and the predicted susceptibility as the longitudinal axis are displayed in Figure 6 to make a quantitative comparison of the magnetic sensitivity accuracy between QSMnet and DIAM-CNN in deep nuclei. Two typical regions of interest, including GP (blue region in Figure 6A) and PUT (red region in Figure 6B), were selected. As shown in Figure 6A, regression slope in GP for DIAM-CNN (slope = 0.961, *R*^2^ = 0.72) was closer to unity than that for QSMnet (slope = 0.94, *R*^2^ = 0.62). Similarly, the regression slope for DIAM-CNN (slope = 0.842, *R*^2^ = 0.73) was closer to unity than that for QSMnet (slope = 0.804, *R*^2^ = 0.65) in PUT, as shown in Figure 6B.

**Figure 7:**
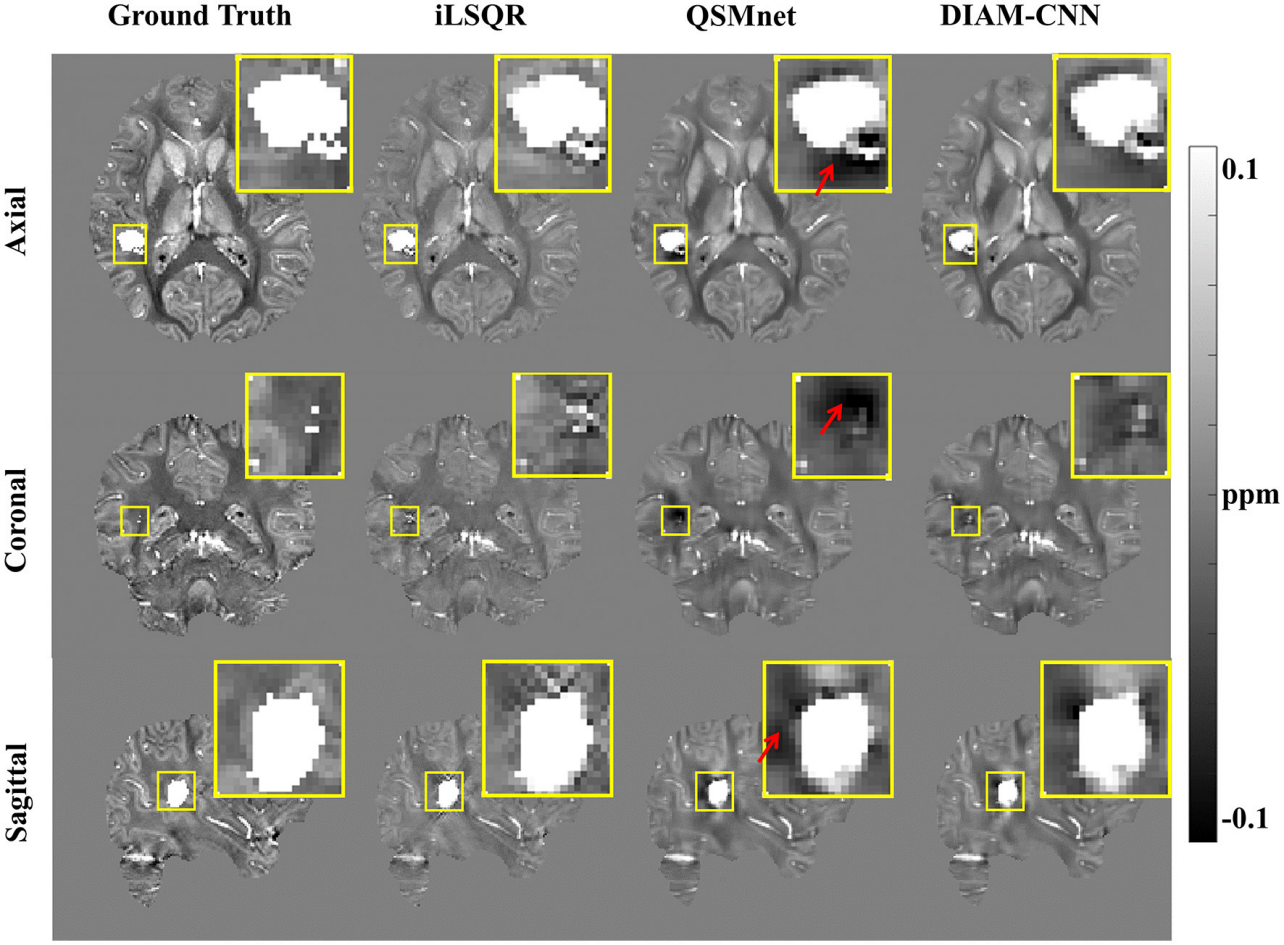
QSM results in the presence of a simulated high-susceptibility ICH lesion. From left to right: QSM maps reconstructed by COSMOS, iLSQR, QSMnet, and DIAM-CNN, respectively. DIAM-CNN outperforms QSMnet in the suppression of shadow artifacts around the lesion, as pointed by the red arrows.

The results of the brain with a simulated high-susceptibility ICH lesion reconstructed by iLSQR, QSMnet, and DIAM-CNN are displayed in Figure 7, with the simulated susceptibility map as the ground truth. In the non-bleeding regions, susceptibility maps reconstructed by iLSQR, QSMnet, and DIAM-CNN showed clear structures. Near the bleeding region, the iLSQR result suffered from severe streaking artifacts, and the QSMnet result showed obvious shadow artifacts, as indicated by the red arrows. In contrast, these artifacts are effectively suppressed in the DIAM-CNN result.

## 4. Discussion

In this study, we proposed a deep learning-based DIAM-CNN method for solving the dipole inversion problem of QSM reconstruction. DIAM-CNN divided the tissue field into high-fidelity and low-fidelity components by thresholding the dipole kernel in the frequency domain and inputs the two components and the original tissue map as individual channels to a multichannel Unet. The results showed that the DIAM-CNN method outperformed QSMnet in terms of quantitative accuracy metrics, including HFEN, NRMSE, PSNR, and SSIM in healthy volunteers. In addition, DIAM-CNN produced fewer artifacts around the high-susceptibility bleeding lesions than the compared methods.

Streaking artifacts in QSM were mainly caused by the noise amplification effect from the division of *D*(*k*) values close to 0 in the dipole deconvolution. Previous studies have shown that streaking artifacts in QSM can be alleviated by particularly processing the low-fidelity component corresponding to small *D*(*k*) values (Wharton et al., 2010). This idea was incorporated by the proposed DIAM-CNN method to construct the neural networks for dipolar inversion, and this may explain the outperformance of DIAM-CNN over QSMnet. The results imply that the characteristics of the problem of QSM reconstruction could be taken into account during the design of the deep-learning QSM method for performance improvement.

The performance of DIAM-CNN depends on the threshold that divides the tissue field into low-fidelity and high-fidelity components. A larger threshold results in more content in the low-fidelity component and less content in the high-fidelity component. A determination of the optimal threshold is time consuming, given the intensive computational burden of network training. In this study, we determined the threshold by a preliminary manual search from 0.1 to 0.5 with an increment of 0.1. The results under varying thresholds showed that a threshold of 0.3 yielded nearly optimal performance for the three-channel DIAN-CNN method. In practice, the optimal threshold may vary with noise levels, and an automatic determination of the optimal threshold can be investigated in future studies.

The proposed DIAM-CNN method has some limitations. First, although DIAM-CNN incorporated the dipolar characteristic of QSM reconstruction only at the stage of input, the design of networks still lacks physical interpretability. The structure of networks and training parameters needs to be tuned empirically. Second, only two components were separated from the tissue field in the current implementation of DIAM-CNN. The tissue field can be separated into more frequency components as more input channels to the network, but computational complexity and GPU memory requirement would significantly increase with the channel number. In addition, determining the optimal choice for multiple thresholds requires a huge computational resource. Third, we experientially set the patch size to 64 × 64 × 64 in the current implementation. Using a larger patch size has the advantage of capturing more global information for QSM reconstruction but with substantially increased computational complexity and GPU memory requirement. Finally, we only combined the dipole-adaptive multiple inputs with the Unet structure. Moreover, the strategy of channel division with dipole-adaptive multi-frequency inputs may be extended to other networks that have shown promising performance in QSM. We will investigate this possibility in future studies.

## 5. Conclusion

This study proposed a deep learning-based method for the dipole inversion in QSM, DIAM-CNN, which adopted a neural network with multiple input channels: the original tissue map, the low-fidelity component, and the high-fidelity component generated by thresholding the dipole in the frequency domain. The experimental results showed that the DIAM-CNN method yielded QSM images with improved accuracy and fewer artifacts than the state-of-the-art methods in both healthy volunteers and hemorrhagic patients. The incorporation of QSM-specific knowledge into the network construction has the potential to improve deep learning-based QSM reconstruction.

## Data availability statement

The original contributions presented in the study are included in the article/supplementary material, further inquiries can be directed to the corresponding author.

## Ethics statement

Written informed consent was obtained from the individual(s) for the publication of any potentially identifiable images or data included in this article.

## Author contributions

WS: methodology, software, validation, and writing. YG: methodology, investigation, data curation, and writing. QZ and JZ: validation. YW: resources and supervision. YF: conceptualization, investigation, resources, writing, supervision, and funding acquisition. All authors contributed to the article and approved the submitted version.
